# Immigrant families' perceptions on walking to school and school breakfast: a focus group study

**DOI:** 10.1186/1479-5868-4-64

**Published:** 2007-12-05

**Authors:** H Mollie Greves, Paula Lozano, Lenna Liu, Katie Busby, Jen Cole, Brian Johnston

**Affiliations:** 1 Department of Pediatrics, University of Washington, Seattle, Washington USA; 2 Seattle Public Schools, Seattle, Washington USA; 3 Feet First, Seattle, Washington USA

## Abstract

**Background:**

Immigrant children face an increased risk of being overweight. Little is known about how immigrant families perceive school programs that may help prevent obesity, such as walking to school and school breakfast.

**Methods:**

Six focus groups (n = 53) were conducted with immigrant parents of school-aged children, two each in three languages: Vietnamese, Spanish, and Somali. A facilitator and translator conducted the focus groups using a script and question guide. Written notes and audio transcripts were recorded in each group. Transcripts were coded for themes by two researchers and findings classified according to an ecological model.

**Results:**

Participants in each ethnic group held positive beliefs about the benefits of walking and eating breakfast. Barriers to walking to school included fear of children's safety due to stranger abductions, distrust of neighbors, and traffic, and feasibility barriers due to distance to schools, parent work constraints, and large families with multiple children. Barriers to school breakfast participation included concerns children would not eat due to lack of appealing/appropriate foods and missing breakfast due to late bus arrival or lack of reminders. Although some parents acknowledged concerns about child and adult obesity overall, obesity concerns did not seem personally relevant.

**Conclusion:**

Immigrant parents supported the ideals of walking to school and eating breakfast, but identified barriers to participation in school programs across domains of the ecological model, including community, institution, and built environment factors. Schools and communities serving immigrant families may need to address these barriers in order to engage parents and children in walking and breakfast programs.

## Background

Childhood overweight disproportionately affects low-income and minority families, including the children of immigrants [1-3]. Immigrants differ from other minority populations in that health status may be initially good, but greater time in the U.S. leads to more obesity and obesity-related behaviors [4-7]. Immigrants now represent the fastest growing minority population in the U.S. [8]; one in every five children in the U.S. is the child of an immigrant. Effective public health interventions are needed to address health concerns for immigrant families, including early intervention to prevent obesity.

The health of immigrant families may be influenced by multiple factors: cultural beliefs, resources, diet and lifestyle [9-13]. Like other low-income families, children of immigrants may face food insecurity and limited access to nutritious foods in their communities [14,15]. They may also have fewer opportunities for safe physical activity and play [16,17].

School-based strategies are recommended to address child overweight, including programs to increase walking to school [18,19] and to promote school breakfast participation [20,21]. The socio-ecological model suggests that such health promotion programs work best when they address underlying beliefs and behaviors that interact with multiple levels of influence in the social environment [22,23]. Given that little is known about developing and promoting school interventions among immigrant communities, this focus group study was undertaken to examine beliefs and barriers among immigrant families for walking to school and school breakfast participation in order to guide development of a school-based obesity prevention program.

## Methods

### Participant recruitment

In summer 2006, six focus groups (n = 53) were conducted with immigrant parents and grandparents of school-aged children from low income neighborhoods in Seattle. Based on a quota purposive sampling frame to obtain representation from different ethnic groups [24], two focus groups were held in each of three languages: Vietnamese, Spanish, and Somali. Participants were recruited through pediatric clinics, community centers, and elementary schools known to serve high proportions of immigrant families. Posters advertising the focus groups were translated into each language. Key stakeholders in each community helped recruit additional participants. Adults eligible for inclusion were foreign-born, spoke one of the study languages as the primary language in the home, and lived with an elementary school child or grandchild.

Written informed consent was obtained from each participant in their language of choice. Participants also completed brief demographic questions. Participants received a meal and child care during the focus groups and a $25 gift certificate at completion. The University of Washington Human Subjects Division approved the study.

### Conducting the focus groups

The project team developed a script and question guide to capture a wide range of beliefs, barriers, and behaviors about 1) walking to school and 2) school breakfast participation, and 3) child obesity. A trained English-speaking facilitator conducted each focus group using standard moderation techniques [25], with a certified interpreter providing simultaneous translation. Open-ended questions were used to start the discussion, followed by questions to elucidate participants' comments. Groups were facilitated in English with simultaneous interpretation and lasted 1.5–2 hours. Audiotapes of the translated discussion were transcribed verbatim. Written notes recorded by a non-participating observer were used to provide more complete contextual analysis when audio transcripts were inaudible.

### Analysis

Two project members (MG, BJ) conducted the theme coding and analysis using data organizing styles previously described [25,26], Transcripts and written notes for each focus group were reviewed for pre-defined themes. The analysis then identified sub-themes within each overarching theme through immersion and multiple re-readings of the transcripts. A codebook developed was used to assign a numerical code for each theme and sub-theme.

The two reviewers independently summarized themes and sub-themes that emerged and reached consensus about discrepancies in interpretation. Coded responses were organized in a Microsoft word document to assess the relative emphasis given to each theme and sub-theme across the ethnic groups [27]. The full project team reviewed, discussed, and agreed upon final interpretation and summary of themes and sub-themes. The socio-ecological model was used to organize the results according to individual/family, community, institutional and environment factors [28].

## Results

Each focus group included 8–12 people, with a total of 17 Vietnamese, 18 Somali, and 18 Spanish speakers. The majority of participants (86%) were female with only one participant per family. Highest education level of participants varied: 45% had grade school or less, 32% had a high school degree, and 10% had a college degree or more, with 13% not responding. Five percent of participants were grandparents. Ninety-five percent of participants had at least 2 children in the home and 41% had 4 or more children. Most participants had lived in the U.S. between 5 and 15 years.

Focus group findings are summarized in Tables 1 and 2 and presented within a socio-ecological model in the Figure. Overall, similar themes emerged across ethnic groups, and results are reported accordingly. Notable findings among parents in a particular ethnic group are indicated in the text.

**Table 1:** Summary of Walking to School Findings

**Domains**	**Themes and sub-themes**
Current practices of school transportation	• Participants–a majority had walked to school in their home country • Participants' children–few walked to school; most driven or bused

Benefits of walking	• Often learned from walking in home country • Physical health benefits including maintaining weight and for preventing heart disease, blood pressure, and diabetes • Mental health benefits–helps children wake up, good for emotions • Being outside in nature, breathing fresh air, seeing natural world • Spending quality time with children, especially to slow down & talk

Barriers to walking to school	**Fear** **Interpersonal** • Fear of abductions; national media stories cited • Bullying, homeless people, gangs • Parents do not know and trust neighbors due to language barriers **Traffic** • Heavy traffic, fast cars • Unsafe street crossings, lack of crossing guards • Lack of walking paths **Feasibility** **Distance/Route** • Many children not living near neighborhood schools • Route too far and/or difficult (e.g. hilly) **Lack of time for parents to supervise walking** • Both parents working (Latino, Vietnamese) • Many families too large for all children to walk together (Somali) • More convenient to drop children off on way to work • Must use child care before/after school • Take bus to school further away to have earlier pick-up time for working parents **Weather/darkness** • Rain, darkness and cold, especially in winter months **Norms** • Lack of necessity to walk; children not accustomed to it

Approaches to improve walking to school	**Street safety** • Improve safety on the route through cross-walks, crossing guards • More safety patrols/police in the neighborhoods • Teach children safety **Facilitate adult-supervised walking (e.g. Walking School Bus)** • Facilitate adults/parents meeting to organize walking school bus • Involve school staff as walking group leaders • More walking after school **Offer more options for children of working parents** • On-site before school child care or supervised walking/activity time

**Table 2:** Summary of Breakfast Findings and Concerns for Child Obesity

**Domain**	**Themes and sub-themes**
Current breakfast practices	• Participants' children typically eat a range of breakfast foods, including both American and culturally traditional foods • Most families did not eat breakfast together during weekdays • Some participants' children ate breakfast at school on occasion

Benefits of eating breakfast	**Good for school performance** • Helps children wake up • Helps children concentrate in school; good as "brain fuel" **Culturally-relevant** • Part of the culture to eat breakfast (Spanish, Vietnamese)

Barriers to eating breakfast in general	**Lack of time** • Families too busy in the morning and children wake up late **Children not hungry** • Ate dinner late • Not active in the morning **Children unaware of the importance of breakfast** • Parents not teaching/reminding children

Barriers to eating school breakfast	**Food content** • Not enough hot dishes, especially culturally-appropriate hot foods • Pork products served (not eaten by Somali families) • Lack of variety **Food quality** • Food too processed and some food expired • Need more fruits and vegetables **Lack of adequate time to eat** • Buses arrive late and children will not get breakfast at all **Concern not adequately supervised** • Children play instead of going to cafeteria • No assurance children will be monitored to eat food served

Approaches to improve school breakfast program	**Food content/quality** • Offer more culturally-specific foods, especially hot main dishes • Survey parents about what they want • Offer taste tests to children **Supervision** • Reminders to children to eat breakfast from school staff/bus drivers **Adequate time to eat** • Ensure bus arrival time appropriate & children get breakfast if late

Concerns for childhood overweight	**Lifestyle in the U.S. worse for physical activity** • More sedentary activities–TV, video games • Parents too busy to supervise children in physical activities • Children living in U.S. less independent, and less fit (Vietnamese) **Concern expressed more about other children, not their own**

### Walking

#### Individual/family attitudes and beliefs about walking

Participants from each language group voiced positive individual beliefs about walking, describing three main benefits: physical and mental health, being close to nature, and spending time with family. Physical health benefits were most frequently cited, including exercise, losing weight, and preventing heart disease and diabetes. Mental health benefits noted across focus groups included that walking is relaxing, enjoyable, an opportunity to slow down, and being "good for depression." Participants highlighted the social benefits of walking with their children: talking, holding hands, and paying more attention to their child, especially compared to driving.

### Barriers to walking to school

Most participants in each language group had walked to school in their country of origin, but only 13% of participants' children walked to school in the U.S. Although parents from each ethnic group expressed support for the idea of walking to school, they identified barriers primarily due to "fear" and "feasibility" over multiple domains of the ecological model.

#### Neighborhood/community

Fear about their children's safety due to threats of violence from strangers was a foremost concern among the groups, as summarized by this parent: "In America it's not safe for young children to walk–too much violence." Parents in every group cited a media story of kidnapping, especially from national media, or a threatened kidnapping near their child's school. As one parent stated, "We hear if kids go by themselves, they'll be kidnapped." Another commented: "In the media, the news on the television, the newspaper, they talk about children being kidnapped." Many parents reported fear of allowing their children to wait at the bus stop unsupervised, let alone walk to school. Some parents described concerns about bullying; others mentioned threats from teenage gangs, homeless people, or drug dealers. Parents also expressed fear and distrust of their neighbors, due in part to language barriers. One Somali group offered another reason: parents may be liable for what happens to their children and fear being criticized for not providing adequate supervision.

#### School/work institutions

Distance and time were primary feasibility barriers to walking to school within the institutions domain. Several participants said their children simply could not walk to school because they lived too far from school: out of necessity due to lack of a neighborhood school or recent school closure, choosing a school perceived to be better, or to be eligible for bus service, sometimes in lieu of before-school care.

Time constraints, especially work start times, prevented many parents from walking with their children. Latino and Vietnamese groups discussed the need for both parents to work to afford living in the U.S. One parent described families in this situation: "the two parents have to work, otherwise, (the family) cannot make it... both parents have to work early, so you cannot walk the kids to school." Mornings were often described as a time of "rushing" to drop kids off at day care or school on the way to work. For Somali parents, having a large family was a barrier to walking with children, with some siblings too young to walk and others attending different schools.

Another barrier identified in each ethnic group was the lack of necessity to walk in the U.S. because of busing policies and car availability, in contrast to their home country where participants described walking regularly for transportation. The lack of necessity to walk influenced the norms of walking among their children. Vietnamese parents specifically described how their children growing up in the U.S. were less active and less independent, resulting in less ability and willingness to walk regularly when they did not have to. Latino parents described their children not wanting to walk until they were told why walking was good for them, or until it became more of a routine: "once they started (walking), they like to do it."

#### Built environment

Traffic danger represented another widely cited fear. Participants noted that streets are wider and traffic volume is greater in the U.S., making crossings unsafe. For example, as one parent described, "Traffic is bad, and my house is close, but they have to pass three stop lights and that's what worries me." Parents noted the unpredictability of drunk drivers and drivers not obeying traffic signals, placing their children at risk even if children are taught traffic safety. Parents also commented on the lack of crossing guards, safe walking routes, and speed control for cars around schools. Several parents across different ethnic groups mentioned a decrease in the number of crossing guards around their children's schools.

#### Natural environment

Parents cited factors in the natural environment as barriers including bad weather, darkness, and hills. As one parent stated, "if the weather is nice, I walk my children to and from school, but if it rains, I drive them."

#### Proposed methods to promote walking to school

Participants were receptive to a "walking school bus" model, with parents or adults leading a group of children walking [29]. Several said they would feel their children were safer if they saw more children walking. Parents described the need to meet other parents to build trust with potential walking group leaders; others mentioned background checks for walking group leaders. Walking after school was suggested as an easier time for some parents to walk with their children. When asked about a "park and walk a block" approach for parents that drive their children [29], most participants were resistant, expressing concern about not having a place to park and cars being stolen or towed. Participants suggested various safety changes including crossing guards, police patrol, and safety training for children and parents. Infrastructure changes identified included building pedestrian paths and a lane around schools to slow cars. Participants also mentioned improving access to neighborhood schools within walking distance and for schools to provide more opportunities for physical activity during the day.

### Breakfast

#### Individual/family attitudes and beliefs about breakfast

Participants held positive beliefs about the benefits of breakfast, especially for children's energy and performance at school. Breakfast was described as being "good for the brain," helping children wake up, concentrate at school, and pay attention to the teacher. Half of participants' children ate school breakfast on some days, with the rest eating at home or day care. Barriers to eating breakfast overall included lack of time, children not being hungry or not being active enough in the morning to have an appetite, and children being unaware of the importance of breakfast. Due to busy or conflicting schedules, most parents did not eat breakfast with their children. Parents reported their children ate mostly "American" foods for breakfast at home (e.g. cereal, waffles, toast, eggs), but also culturally-specific foods, (e.g. Vietnamese: noodle soups, sticky rice; Latino: tortillas, quesadillas; Somali: injera bread, halal meat).

### Barriers to school breakfast participation

#### Community

Somali families cited religious prohibitions against eating pork as a barrier for their children eating at school. Participants in Vietnamese and Spanish groups mentioned that breakfast was an important part of their culture.

#### Institutions

Concerns expressed about school breakfast food focused primarily on the quality, palatability, and lack of variety. Some parents cited lack of freshly prepared foods at school and some cited poor food quality, such as expired milk. Several parents expressed concern their children did not like some foods and then would not eat. A concern mentioned in Latino and Vietnamese groups was a lack of hot main dishes served for breakfast; hot food was "better for the stomach" as expressed by a Vietnamese parent. A Latino parent described that, because her daughter's school brings prepared food from somewhere else, "they hardly ever have a warm breakfast... it's not home style. So they (the school staff) don't make breakfast taking into consideration the children's background." Several parents considered cold cereal, which they perceived to be the main item available at school breakfast, inadequate on a daily basis. Only the Latino parents cited nutrition concerns, mentioning lack of fresh fruits and vegetables, too much processed food, and too much juice.

Several parents in different groups expressed concern about children missing breakfast at school due to bus late arrival and lack of adult supervision to remind children to go to the cafeteria and to ensure adequate intake. For example, several parents said their children would go to the playground instead of the cafeteria and then miss breakfast: "As soon as they get to school, they'd rather play than eat." Other parents expressed concern that their children would not finish breakfast without adult supervision.

#### Proposed methods to promote breakfast

Parents recommended improving school breakfast quality with more variety, hot main dishes, and culturally-diverse foods, although foods suggested varied across cultural groups. One parent suggested that parents and families should be surveyed about what they would like to have served. Another parent offered the idea to provide taste tests for children. Parents also thought school staff should remind children to eat breakfast. Bus arrival needed to be early enough to allow children to eat and if school buses were late, parents wanted to be sure children would still be able to eat breakfast.

### Concerns about child obesity

In general, parents did not endorse the relevance of child obesity as a personal concern. Many parents stated that their own children, if anything, were too thin, not overweight. Only two parents in all the groups acknowledged concern about their own child being overweight. A few parents expressed concern about their own weight gain following lifestyle changes and reduced walking after moving to the U.S. Parents did recognize child obesity as a problem overall in the U.S., citing more TV, video games, less activity, and less time for parents to supervise children's active play. For example, one Vietnamese parent described overweight as a problem because of "the environment we live in–they play too many video games, they watch too much TV, they're not moving their bodies."

## Discussion

Immigrant parents of elementary school children from diverse backgrounds held positive beliefs about the benefits of walking to school and eating breakfast. Despite supportive individual beliefs, participants identified significant barriers to participation in walking to school and school breakfast programs across community/neighborhood, institutional and built/natural environment domains described in the socio-ecological model [22,28]. These barriers demonstrate the numerous, potentially conflicting, influences on behavior, but also suggest multiple possible avenues for intervention.

For walking to school, predominant barriers among immigrant families emerged from within the community/neighborhood (kidnapping) and built environment domains (traffic safety) that elicited fear among parents for their children's safety. Findings from the present study are consistent overall with barriers to walking to school identified among non-immigrant families in the U.S. (traffic safety, distance, crime) [30], and in Australia (traffic safety, poor street crossings, distance) [31]. Kerr et al. found that level of parental concern was the strongest explanatory factor for likelihood of children walking or bicycling to school [32] and Timperio et al. reported that concern about strangers was universal to parents in terms of child safety for walking and bicycling [33]. Immigrant families seemed to place greater emphasis on the danger of child abductions relative to traffic injuries, which could be explained by distrust of neighbors due to language and other cultural barriers, which parents reported, and to living in low-income neighborhoods. Indeed, Weir et al. found associations between parent neighborhood fears with lower physical activity among children in poor urban neighborhoods [34]. Focus group participants in this study frequently cited the media in highlighting abduction dangers, suggesting that immigrant parents depend on media for learning information about life in the U.S. [35] and may be more susceptible to alarming news reports.

The implications of the present findings for walking to school suggest several avenues for program development among immigrant families. As previously advocated by McMillan et al., [36], this study highlights the need to address both real and perceived barriers, such as objective built environment changes (crossing guards/signals, walking paths) and perceptions that engender fearfulness (media reports about abductions). First, programs within immigrant communities should highlight the cultural relevance and underlying value about the benefits of walking, as developed through experience walking in their home country. Second, while immigrant parents were receptive to the walking school bus model [29], language barriers and parent work schedules necessitate support from schools and community-based organizations to facilitate families meeting and building relationships to share supervision. Approaches might include walking in the afternoon when fewer parents work or using school staff as walking group leaders. Third, institutions could provide comprehensive access to school walking programs to working parents, such as offering walkable on-site before school care or activities, or physical activity programs once children arrive. Fourth, in addition to teaching safety to children, participants recommended improved safety supports and built environment design, such as the use of crossing guards, and structural improvements like pedestrian paths and traffic slowing around schools. There is evidence that such built environment changes can result in higher rates of walking to school [37].

In relation to breakfast, parents in all three ethnic groups described the importance of breakfast consumption for children's performance in school, which is consistent with findings reported in the literature from breakfast programs [38-40]. Although breakfast consumption has been associated with lower risks of child overweight [21,41], none of the parents in this study mentioned weight control in relation to breakfast. For school breakfast participation, primary barriers emerged from within the community and institutional domains of the ecological model. Time scarcity, which has been identified as a barrier to eating nutritious meals among low-income families [42], was frequently described by parents in this study about their morning routines. Promoting school breakfast may therefore be especially helpful to these families to provide convenient, nutritious meals; however, parents' concerns need to be addressed. As for walking to school, the inability to supervise children was an important barrier among immigrant parents to support school breakfast. Similar to prior research among non-immigrant families, school bus arrival was a concern for immigrant parents about children not getting enough time for breakfast [43]. Additional concerns not previously identified among non-immigrant populations included inadequate variety of culturally-appropriate breakfast foods (particularly hot meals), inadequate adult supervision during breakfast, and lack of reminders for children to eat breakfast.

The findings about school breakfast highlight the need for improved communication with immigrant families about school breakfast programs, including the hot and cold options available daily, efforts to ensure that school meals meet children's religious or other dietary restrictions, and the availability of adult supervision provided to ensure that children are reminded to get breakfast in the cafeteria and actually consume the food provided. The limited financial resources for school meals make providing appealing, nutritious foods in ethnically diverse schools a challenge. School meal programs may benefit from actively engaging children and families, such as through taste tests and parent surveys. Lengthening the time between bus arrival and the start of classes may also be warranted to provide children adequate time to arrive and consume breakfast at school.

Based on results of this study, preventing child obesity should not necessarily be considered a primary strategy to promote school programs among immigrant families. Parents tend to underestimate their children's weight [44-46], which may partly explain why few participants in this study expressed concern about their own children's weight. While participants acknowledged the overall problem of child obesity, child obesity prevention did not emerge as a personally motivating factor among immigrant parents. Instead, having energy and nourishment for school performance were cited as positive factors for walking to school and eating breakfast.

Limitations of this study include the small number of focus groups conducted in each language, which limits the extent of comparative analysis possible between the three ethnic groups studied. The design of this study, however, was to explore overall themes among immigrant parents rather than to draw specific conclusions about each ethnic group. A limitation of focus group research, in particular, is the challenge of discussing sensitive topics [25], such as cost of meals, which was *not *identified as a barrier to participation in school meals by parents in this study, although cost has been described as a factor in previous studies [43]. Unlike quantitative methods, which uses probability sampling, sampling in qualitative methods, as used here, seeks to provide "information-rich" cases [47]. Thus, results here are not intended to be statistically representative. Results of this study can, nonetheless, inform the perspective of those working with immigrant parents to enrich the understanding of cultural and personal factors relevant to promoting school-based physical activity and meals programs [48].

## Conclusion

**Figure 1:**
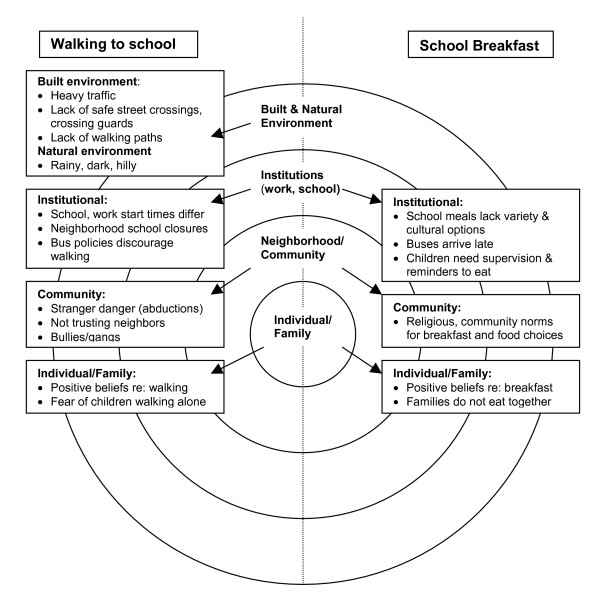
Socio-ecological model presenting barriers to walking to school and school breakfast participation among immigrant families.

Using an ecological framework to elucidate barriers in multiple environments, this is the first study reporting on walking to school and school nutrition program participation specific to immigrant families. Predominant themes for both walking and eating breakfast include immigrant parents' fears about child vulnerability in the new country and need to supervise their children and ensure their safety and welfare (see Figure 1). Schools and community programs working with immigrant families can draw on the assets of strong parent support expressed for walking and eating breakfast. Immigrant families identified need for institutional and community changes, as well as broader environment changes, to facilitate participation in school programs that promote walking to school and school breakfast.

## Competing interests

The author(s) declare that they have no competing interests.

## Authors' contributions

MG participated in the design, coordination and carried out the initial data analysis and drafted the manuscript. PL contributed to the design of the figure and drafting and review of the manuscript. LL participated in the design of the study, review of data analysis and final interpretation of results. JC and KB participated in the data collection, review of data analysis and final interpretation of results. BJ conceived of the study, participated in its design, coordination and data analysis, and helped to draft the manuscript. All authors read and approved the final manuscript.
